# Environmental parameters and microbial community profiles as indication towards microbial activities and diversity in aquaponic system compartments

**DOI:** 10.1186/s12866-020-02075-0

**Published:** 2021-01-06

**Authors:** Zala Schmautz, Carlos A. Espinal, Andrea M. Bohny, Fabio Rezzonico, Ranka Junge, Emmanuel Frossard, Theo H. M. Smits

**Affiliations:** 1 Ecological Engineering Centre, Institute of Natural Resource Sciences, Zurich University of Applied Sciences, Wädenswil, Switzerland; 2 Group of Plant Nutrition, Institute of Agricultural Sciences, ETH Zurich, Lindau, Switzerland; 3 Landing Aquaculture, Boxtel, The Netherlands; 4 Environmental Genomics and Systems Biology Research Group, Institute of Natural Resource Sciences, Zurich University of Applied Sciences, Wädenswil, Switzerland

**Keywords:** Aquaponics, Chemical analysis, Community analysis, Archaea, Bacteria, T-RFLP

## Abstract

**Background:**

An aquaponic system couples cultivation of plants and fish in the same aqueous medium. The system consists of interconnected compartments for fish rearing and plant production, as well as for water filtration, with all compartments hosting diverse microbial communities, which interact within the system. Due to the design, function and operation mode of the individual compartments, each of them exhibits unique biotic and abiotic conditions. Elucidating how these conditions shape microbial communities is useful in understanding how these compartments may affect the quality of the water, in which plants and fish are cultured.

**Results:**

We investigated the possible relationships between microbial communities from biofilms and water quality parameters in different compartments of the aquaponic system. Biofilm samples were analyzed by total community profiling for bacterial and archaeal communities. The results implied that the oxygen levels could largely explain the main differences in abiotic parameters and microbial communities in each compartment of the system. Aerobic system compartments are highly biodiverse and work mostly as a nitrifying biofilter, whereas biofilms in the anaerobic compartments contain a less diverse community. Finally, the part of the system connecting the aerobic and anaerobic processes showed common conditions where both aerobic and anaerobic processes were observed.

**Conclusion:**

Different predicted microbial activities for each compartment were found to be supported by the abiotic parameters, of which the oxygen saturation, total organic carbon and total nitrogen differentiated clearly between samples from the main aerobic loop and the anaerobic compartments. The latter was also confirmed using microbial community profile analysis.

**Supplementary Information:**

The online version contains supplementary material available at 10.1186/s12866-020-02075-0.

## Background

The prevailing microbial interactions occur as a result of the association of microorganisms with a surface [1]. These associations, known as biofilms, are most often microbial communities harboring bacteria, archaea, unicellular eukaryotes and fungi [2]. Depending on the dominant environmental parameters, such as nutrient and oxygen availability, hydrodynamics and microbial composition, the location and structure of the biofilm are changing [1–3].

In an aquaponic system, which is a combination of recirculating aquaculture system and hydroponics, microbial communities and their metabolic products play a vital role in various molecular processes. These processes include the transformation of nitrogenous compounds, the consumption of organic matter, the mineralization of complex organic molecules [4], the consumption of dissolved oxygen, the production of carbon dioxide, the consumption and replenishment of water alkalinity [5]. These processes are important, as they all directly affect plant development and the welfare of the fish grown in such systems. Microbes transform fish metabolites into compounds that plants use for their growth [6], and thus, they are essential for the proper functioning of the system [7].

The environmental requirements of all involved organisms (microorganisms, plants and fish) are species- and developmental-stage specific. Therefore, the cultivation conditions should ideally reflect this appropriately [8–13]. Besides the main abiotic parameters such as temperature, pH and oxygen saturation, nutrients and more specifically, nitrogen (N) and carbon (C) play a major role in the performance of different aquaponic system compartments [14]. Nitrogen is present either in its organically-bound form (N_org_) in cellular and extracellular compounds [15] or in its inorganic forms (ammonium: NH_4_^+^, nitrite: NO_2_^−^ or nitrate: NO_3_^−^) and acts as a source for microbial metabolic processes [16], from which the products, NH_4_^+^ and NO_3_^−^, are further used as a nutrient source for the plants [17]. Alongside N, C in its inorganic form, carbon dioxide, is used for photosynthesis [18], while organic C forms the largest C-pool in the water [19]. Furthermore, the ability of the C-pool to bind nutrients can affect primary production in an aquatic environment [20] as specific microbial populations can utilize both organic C and inorganic C through heterotrophic, chemoautotrophic, and photoautotrophic pathways under aerobic, anaerobic, and anoxic conditions [5].

**Fig. 1:**
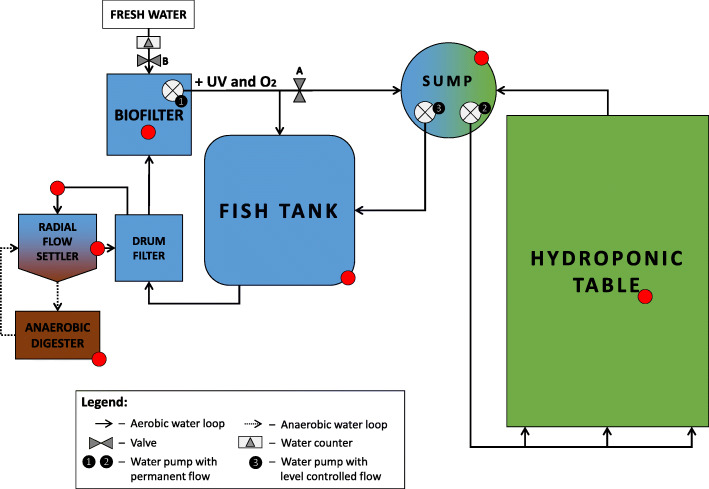
Water flow in one replicate of three aquaponic systems as operated between 2017 and 2018 with an anaerobic (marked brown) and aerobic loop consisting of an aquaculture (marked blue) and hydroponic component (marked green), and sampling points (marked with red dots): Using gravity, water from the fish tank was continuously flowing through the solids removal unit to the biofilter. In the solids removal unit i.e. drum filter, the solids (fish feces and feed residues) were mechanically separated from the clear system water with a 40 μm mesh drum filter. A circulation pump (1) was continuously (5 m^3^ h^−1^) pumping water from the biofilter through the UV and oxygenation zone. The computer-controlled valve B, opened every 5 min for 2 min, resulting in a water flow of 0.5 m^3^ h^− 1^ to the sump. A level sensor-controlled pump (3) then pumped the water back to the fish tank keeping the water level in the sump stable. A different pump (2) was continually pumping (0.36 m^3^ h^− 1^) the water to the hydroponic raft table and from there back to the sump over the drainage point. To maintain a constant water level and to control water consumption in the system, fresh tap water was added to the system via a mechanically controlled water valve and analogous water counter. During the automatic drum filter rinsing with clear system water, small amounts of water with solids were rinsed into the solid thickening unit i.e. radial flow settler. Three times per week, 7 L of thickened settled sludge was manually removed and added to the anaerobic digester, at the same time, 7 L of the supernatant from anaerobic digester was added back to the radial flow settler, which returned water full of nutrients to the main water loop of the system

Due to the design, function and configuration of the aquaponic systems, each of the individual compartments presents different environmental conditions (Fig. 1, Table 1) [6, 21]. These conditions will shape the microbial processes occurring in the compartments, and thus, affect the quality of the water being passed through them. Since finding the balance between the requirements for fish, plants and microorganisms is important for a successful aquaponic production system [22], fish welfare and plant vitality, understanding how different compartments may affect this balance, becomes crucial. Presently, a correlation between the compartment dependent abiotic parameters and microbial community structure has yet to be studied in these systems.

**Table 1:** Compartments of the aquaponic system, their functions, targeted water parameters and expected reactions in each compartment

Compartment	Function	Targeted parameters	Expected reactions
**Fish tank** (aerobic loop)	Fish holding	Dissolved oxygen close to or at saturation, low total nitrogen, total organic carbon, NH_4_^+^ and NO_2_^−^	Nitrification, aerobic respiration
**Drum filter** (aerobic loop)	Removal of the solids via mechanical filtration	Low suspended solids after the filtration	None
**Biofilter** (aerobic loop)	Oxidation of total ammonia nitrogen and nitrite to nitrate	Dissolved oxygen close to or at saturation, low NH_4_^+^ and NO_2_^−^	Nitrification, aerobic respiration
**UV treatment** (aerobic loop)	Water disinfection	None	None
**Oxygenation zone** (aerobic loop)	Saturation of the system water with oxygen	Dissolved oxygen at saturation	None
**Sump** (aerobic loop)	The connection between aquaculture and hydroponic part of the system, serving as a hydraulic buffer	High oxygen saturation, high NO_3_^−^ and low NH_4_^+^ and NO_2_^−^	Nitrification
**Hydroponic table** (aerobic loop)	Plant holding on a thin layer of water with floating polystyrene foam rafts	High oxygen concentration, high NO_3_^−^_,_ low NH_4_^+^ and NO_2_^−^	Nitrification, plant nutrient uptake, carbon input by rhizodeposits, microbial respiration
**Radial flow settler** (aerobic and naerobic loop)	Passive filtration by using gravity to remove settable solids	Inflow: High concentration of suspended solids, total organic carbon and total nitrogen, higher NH_4_ compared to the outflow Outflow: Reduced suspended solids and TN compared to the inflow	Nitrification, denitrification, ANAMMOX, microbial fermentation, mineralization
**Anaerobic digester** (anaerobic loop)	Anaerobic sludge digestion to obtain supernatant rich with nutrients and recycle the water	High nutrient concentration, no/low oxygen, low redox potential, high solids, accumulation of NH_4_	Denitrification, acidogenesis, hydrolysis, iron and sulfate reduction, sulfate oxidation, carbon mineralization, methanogenesis, dissimilatory nitrate reduction

Using the aquaponic systems located in Wädenswil, Switzerland, we determined water parameters and microbial community profiles (archaea and bacteria) in biofilms from different system compartments with the aim to obtain first data to support the predicted metabolic processes taking place in the system and to investigate which of the abiotic parameters may be correlated to the microbial diversity in each compartment. While all compartments showed unique abiotic parameter levels, the overall results imply that the compartment design and mode of operation rather shape the specific microbial community composition.

## Results

Three parallel aquaponic systems (Fig. 1), planted with a mix of herbs and stocked with tilapia (*Oreochromis niloticus)* at a density of 10 kg m^− 3^ were set up in May 2017 and operated continuously. At the time of both samplings, in September 2018 (Table 2), all three systems showed a steady performance based on the water quality measurements (Table 3).

**Table 2:** Mean ± SEM for taxonomic richness presented as operational taxonomic units (OTUs) of bacterial and archaeal biofilm communities from different compartments of an aquaponic system analyzed with terminal restriction fragment length polymorphism. No significant differences were found between the estimates of taxa richness per sampling date and different sampling places. Numbers in the brackets present the number of analyzed samples^*a*^

	Sampling date	Fish tank	Biofilter	Sump	Hydroponic table	RFS inflow	RFS outflow	Anaerobic digester
**Bacteria**	Week 37 11.9.2018	104.2 ± 5.7 (18)	101.1 ± 5.2 (8)	95.3 ± 8.3 (9)	92.8 ± 7.9 (9)	92.1 ± 8.3 (8)	86.6 ± 7.1 (9)	84.4 ± 6.1 (9)
	Week 39 26.9.2018	153.6 ± 5.9 (18)	149.3 ± 9.1 (8)	150.6 ± 5.6 (9)	152.3 ± 7.6 (9)	161.7 ± 5.2 (9)	137.4 ± 8.7 (9)	133.0 ± 10.1 (9)
**Archaea**	Week 37 11.9.2018	5.7 ± 1.1 (7)	14.3 ± 5.8 (3)	2.8 ± 0.4 (5)	1.5 ± 0.5 (2)	5.2 ± 2.3 (4)	8.7 ± 4.1 (3)	9.0 ± 1.4 (4)
	Week 39 26.9.2018	25.1 ± 2.4 (18)	22.2 ± 3.3 (6)	26.6 ± 2.9 (9)	28.5 ± 3.7 (8)	26.6 ± 2.8 (9)	28.8 ± 2.9 (9)	20.5 ± 6.5 (2)

^*a*^ analytical replicates of three experimental replicates

**Table 3:** Mean ± SEM of water quality parameters in different compartments of an aquaponic system combining both sampling times. Letters present the significant differences between the compartments of the system based on Kruskal-Wallis rank sum test followed by Fisher’s LSD test (α = 5%, *n* = 6)

	Fish tank	Biofilter	Sump	Hydroponic table	RFS inflow	RFS outflow	Anaerobic digester
**Temp** [°C]	26.9 ± 0.2ab	27.9 ± 0.3a	25.7 ± 0.5ab	25.1 ± 0.5b	25.4 ± 0.6b	24.9 ± 0.6b	26.1 ± 1.1ab
**pH** [−]	7.20 ± 0.03ab	7.33 ± 0.07a	7.27 ± 0.02a	7.24 ± 0.05a	7.09 ± 0.03b	7.08 ± 0.03b	7.34 ± 0.07a
**EC** [μS cm^−1^]	1748 ± 65b	1743 ± 65b	1731 ± 64b	1721 ± 68b	1720 ± 71b	1716 ± 67b	2984 ± 42a
**Oxygen** [%]	107.7 ± 3.4a	99.2 ± 1.5a	101.9 ± 5.4a	80.7 ± 6.6b	41.5 ± 8.5c	36.1 ± 3.8c	1.8 ± 0.2d
**Redox** [mV]	105.4 ± 12.5a	112.9 ± 13.8a	94.8 ± 13.4a	99.9 ± 12.9a	69.1 ± 20.2ab	86.2 ± 15.7a	− 298.8 ± 8.0b
**TOC** [mg L^−1^]	46.9 ± 3.6d	48.1 ± 3.6d	99.9 ± 4.5c	39.7 ± 2.0d	357.6 ± 200.8bc	131.6 ± 8.5b	3211.2 ± 258.2a
**TN** [mg L^−1^]	129.5 ± 9.1bc	105.5 ± 11.7c	133.4 ± 7.9bc	138.9 ± 7.7b	135.3 ± 8.2ab	119.2 ± 7.1bc	613.7 ± 50.3a
**NH**_**4**_^**+**^**-N** [mg L^−1^]	0.23 ± 0.01bc	0.14 ± 0.03 cd	0.10 ± 0.01d	0.08 ± 0.01d	0.80 ± 0.51b	0.27 ± 0.07bc	215.06 ± 18.86a
**NO**_**2**_^**−**^**-N** [mg L^−1^]	0.05 ± 0.01c	0.06 ± 0.01bc	0.06 ± 0.01c	0.02 ± 0.00d	1.63 ± 0.57a	1.38 ± 0.39ab	0.08 ± 0.03 cd
**NO**_**3**_^**−**^**-N** [mg L^−1^]	132.8 ± 11.5bc	125.4 ± 10.2c	158.8 ± 4.7ab	180.9 ± 16.2a	135.23 ± 12.7abc	129.4 ± 18.3bc	0.5 ± 0.0d
**Ca**^**2+**^ [mg L^−1^]	94.4 ± 4.4a	79.8 ± 3.2ab	65.6 ± 9.7ab	63.3 ± 7.7b	79.7 ± 4.5ab	81.1 ± 8.2ab	76.2 ± 4.5ab
**Mg**^**2+**^ [mg L^−1^]	27.1 ± 1.2a	25.1 ± 0.8a	20.7 ± 3.4a	21.9 ± 4.0a	23.9 ± 1.0a	23.7 ± 1.6a	27.2 ± 2.0a
**Na**^**+**^ [mg L^−1^]	207.9 ± 13.4a	213.1 ± 20.0a	175.6 ± 33.6a	149.4 ± 18.5a	172.5 ± 12.9a	175.5 ± 12.0a	164.6 ± 13.8a
**K**^**+**^ [mg L^−1^]	60.9 ± 11.6a	93.8 ± 25.4a	70.9 ± 21.4a	52.9 ± 19.8a	36.5 ± 4.0a	55.4 ± 21.7a	68.7 ± 10.7a
**SO**_**4**_^**2−**^**-S** [mg L^−1^]	200.9 ± 42.1a	196.2 ± 14.9a	183.8 ± 63.4a	161.3 ± 59.2a	195.4 ± 53.2a	173.6 ± 41.3a	20.0 ± 0.3b

### Microbial community profiles

To obtain insight into archaeal and bacterial community profiles, biofilm samples from different compartments of the aquaponic system (Fig. 1) were taken and analyzed using terminal restriction fragment length polymorphism (T-RFLP), done separately for bacterial and archaeal communities. Biofilm samples showed differences in community structure and operational taxonomic unit (OTU) abundance between different compartments of the system. There was a difference in the number of observed OTUs between the two sampling dates for both, bacteria and archaea. Since the results for both sampling dates showed the same trend (Additional file 1: Table S1 and Figure S1), data were combined for further analyses.

**Fig. 2:**
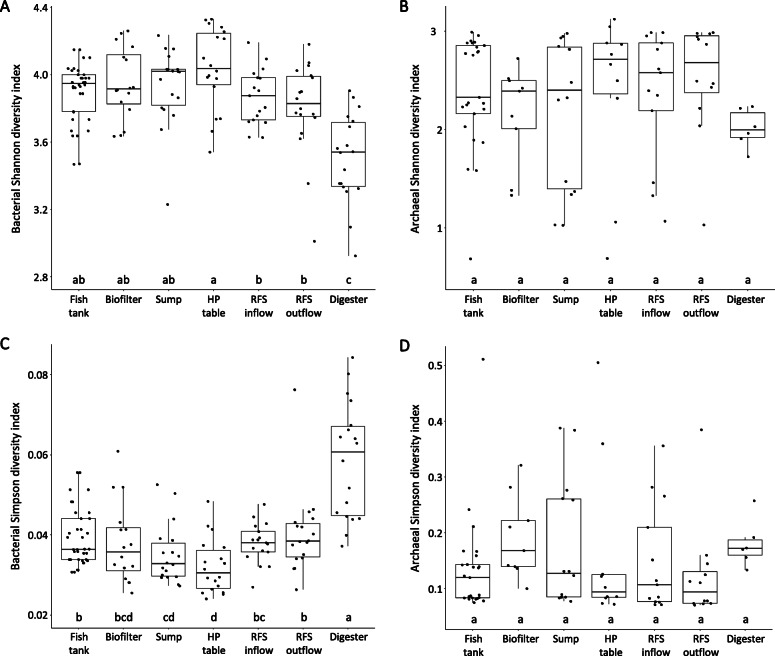
Shannon and Simpson diversity indices for bacteria (**a**, **c**) and archaea (**b**, **d**) based on the relative abundance of terminal restriction fragment peak area combined for both sampling times. Different letters are indicating significant differences between the compartments (fish tank, biofilter, sump, hydroponic table, radial flow settler inflow and outflow, and anaerobic digester) of the aquaponic system as shown on Fig. 1 (marked with red dots) based on Kruskal-Wallis rank sum test followed by Fisher’s LSD test (α = 5%)

No differences in the taxa richness could be shown between samples from individual compartments (Table 2). However, bacterial data showed higher Shannon diversity, while archaeal data showed higher Simpson diversity, indicating lower diversity due to dominating OTUs within the archaeal community (Fig. 2, Additional file 1: Table S1). Biofilm samples from the anaerobic digester stood out compared to the other compartments. Here, the T-RFLP data suggested a lower taxa richness, as shown by the Shannon and Simpson diversity indices. With lower diversity and higher dominance than the rest of the aerobic part of the system, the bacterial biofilm from the solids thickening unit (radial flow settler – RFS), the connection between aerobic and anaerobic conditions, was more similar to that of the anaerobic digester.

Bacterial diversity was highest in the biofilm from the hydroponic table (Fig. 2). This effect on the microbial biodiversity can be caused by the potential influence of various herbs planted in the system, as each plant species enriches its unique root microbiome [23, 24]. The biofilm of the sump, which serves as a connection between aquaculture and hydroponic part of the system, showed a high diversity as well, which could be the result of the influence of both connecting environments, and therefore, indicative for the presence of OTUs originating from both, aquaculture and hydroponic environment.

**Fig. 3:**
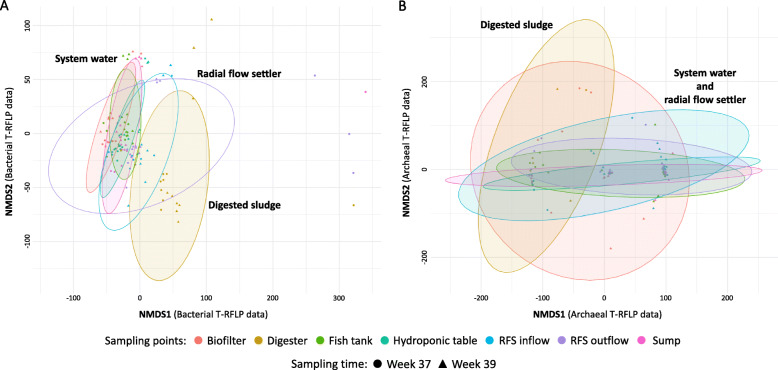
Non-metric multidimensional scaling analysis of bacterial (**a**, ADONIS *R*^2^ = 0.297, dimensions = 2, stress = 0.157) and archaeal (**b**, ADONIS *R*^2^ = 0.141, dimensions = 2, stress = 0.129) communities in different compartments (fish tank, biofilter, sump, hydroponic table, radial flow settler inflow and outflow, and anaerobic digester) of the aquaponic system with 95% confidence eclipses combining data of both sampling times

The bacterial community clearly differed between the aerobic and anaerobic compartments of the aquaponic system (Fig. 1, Table 1), which can be seen from the non-metric multidimensional scaling (NMDS) based on the Manhattan distance (Fig. 3, Additional file 1: Figure S1). The communities in the RFS, which serves as a connection between the aerobic part of the system and anaerobic digester, appeared to be influenced by both aerobic and anaerobic environment. The OTU distribution within the RFS overlapped between the aerobic and anaerobic condition clusters. Contrarily to the bacterial community, the archaeal community showed no clear separation between the compartments.

### Abiotic parameters

Parallel to the microbial biofilm samples, water samples were taken from the same location in the compartments (Table 3). Water temperature in the different system compartments reflected the direction of circulation flow as well as the source of the heat input. The highest temperatures occurred in the biofilter where the heat exchanger for cooling and heating was installed. Additionally, the water in the biofilter had contact with warm air coming from the diffused aeration system ensuring proper mixing of the water and biofilter media. Lowest temperatures were present in the hydroponic table and RFS, which can be explained by these compartments having increased surface exposure to the greenhouse environment, transpiration by plants and holding relatively small volumes of water. The rest of the system showed stable temperatures over time and between the compartments.

Electrical conductivity did not vary significantly between the aerobic compartments of the system. Only in the anaerobic digester, the electrical conductivity was significantly higher compared to the rest of the system, most probably, due to the release of organically bound ions via mineralization [25].

Oxygen saturation and redox potential in the different compartments should reflect the processes of oxygen supply and consumption. This revealed the highest saturation in the fish tank since the water comes directly from the oxygenation device (low head oxygenator). The hydroponic table had significantly lower oxygen compared to the fish rearing compartment. As the water film is relatively thin and a large surface area is available, it would be expected that sufficient saturation would be achieved. However, as the opposite was found, this would suggest that a high microbial activity and root respiration were reducing the oxygen saturation to 80%. In contrast to the rest of the system, the oxygen saturation in the anaerobic digester was below 2% and redox potential between - 319.4 mV and - 278.3 mV, confirming anaerobic conditions.

Values of pH were all within the targeted range (pH ≈ 7) and did not differ between the compartments. However, compared to the rest of the aerobic part of the system, the pH values in the RFS were lower at both time points. Likely, there was incomplete oxidation of the C substrates from the captured sludge, which led to lower oxygen saturation and acidification of the water from this compartment based on fermentation activity.

### Total organic carbon and total nitrogen

Total organic carbon (TOC) and total nitrogen (TN) of water samples were analyzed to investigate changes in organic matter, which showed apparent differences between compartments of the aquaponic system. The highest TOC levels were found in compartments where solids tend to accumulate (anaerobic digester, RFS and sump).

In contrast to TOC, TN is vital for the nitrogen assimilation into cell compounds [15] or as a substrate for degradation to inorganic N forms [26]. This implies that higher TN levels are expected in compartments where higher concentrations of organic matter are found (i.e. hydroponic table, RFS and anaerobic digester). In contrast, low TN concentrations were found in the biofilter and fish tank, where organic matter and biofilms are continuously removed by a combination of tank flushing and periodic mechanical cleaning.

### Other nutrients

Besides N and C, plants and microorganisms depend on other macro- and micronutrients that can be limiting factors for their growth [18]. As the concentration of certain nutrients within the same water column may correlate to one another, we measured them in different compartments of the aquaponic system (Table 3). Although not significant, concentrations showed a trend that reflected the direction of nutrient flow through the aquaculture part, where the fish feed entered the system, to the hydroponic part, indicating nutrient uptake by the plants [18, 27].

### Influence of environmental parameters on community structure

**Fig. 4:**
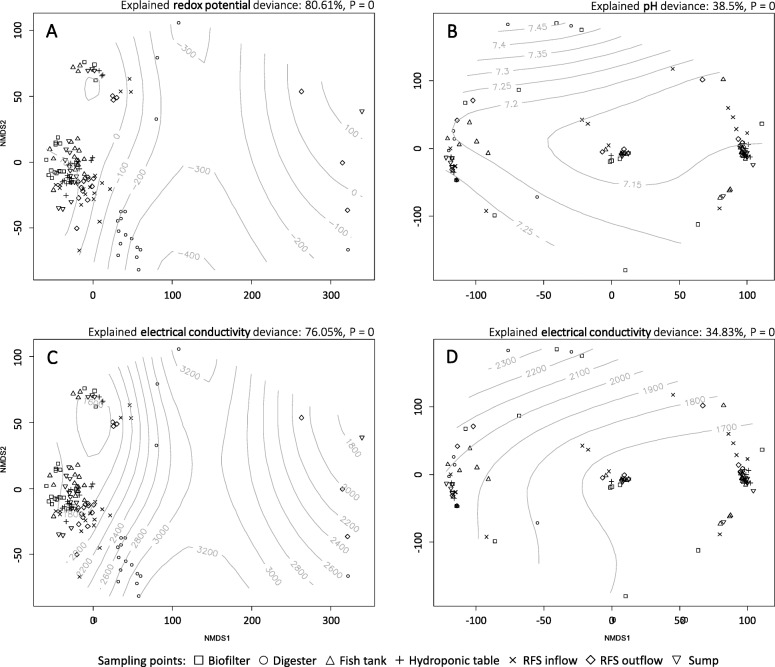
Non-metric multidimensional scaling (NMDS) plot of bacterial (**a**, **c**) and archaeal (**b**, **d**) communities with generalized additive models (gray lines) of two most explanatory environmental variables

The principal component analysis of the main indicator parameters (Additional file 1: Figure S2) showed that abiotic parameters clearly differ between compartments. In total, more than 82% of the variance was explained. To assess the influence in habitat preferences of the microbial community, the measured abiotic parameters were merged with the terminal restriction fragment abundance matrix and plotted in 2-dimensional figures separately for bacteria and archaea (Fig. 4, Additional file 1: Figure S3 and Figure S4). Combining abiotic parameters with microbial data was able to explain up to 80 and 38% of variability, for bacteria and archaea, respectively. Electrical conductivity showed a strong influence on both, bacterial and archaeal communities, while other abiotic parameters differed between the communities. Strong influence of environmental parameters was also shown by using redundancy analysis (Additional file 1: Figure S5A), separating the bacterial community into two clusters, aerobic and anaerobic cluster. Electrical conductivity, TOC and TN had a strong influence on the anaerobic cluster, while redox potential and oxygen saturation rather influenced the aerobic cluster. Redundancy analysis of the archaeal community did not show any significant effect of the environmental parameters (Additional file 1: Figure S5B).

## Discussion

Each compartment of the aquaponic system has a specific function with its own distinct environmental conditions (Table 1), shaped by system operation as well as by the presence of the different organisms (fish, plants and microorganisms). The main differences were observed between aerobic (fish tank, biofilter, sump and hydroponic table) and anaerobic (anaerobic digester) part of the system, with the RFS connecting both loops. The aerobic and anaerobic loops strongly differed in electrical conductivity, redox potential, oxygen saturation, TOC and TN (Table 3).

High and positive redox potential is indicative of oxic conditions, as seen in the aerobic part of the aquaponic system (Table 3), whereas a low and negative redox indicates a strongly reducing environment [28]. A comparison to the processes in geochemical cycles would suggest that anaerobic reactions, such as denitrification (starting at 100 mV, but strictly anaerobic), sulfate reduction (at − 100 mV) and methanogenesis (at − 150 mV to − 400 mV) could occur under the conditions found in this reactor [29, 30]. Since sulfate reduction, as observed by a lower sulfate concentration in the anaerobic reactor (Table 3), only occurs when iron is completely reduced [31], future assessment of iron concentrations in the digesters would thus assist with the evaluation of sulfate reduction in the system.

The accumulation of TOC in the anaerobic reactor was accompanied by primary depletion of oxygen (Table 1), which may have led to the production of NH_4_^+^ through dissimilatory NO_3_^−^ and sulfate reduction (Table 3), both processes known to occur in anaerobic reactors with excess C [32]. A higher TOC concentration in the RFS compared to the other aerobic compartments corresponds to the accumulation of solids in this compartment. In this compartment, the accumulation could have resulted in increased heterotrophic activity, shown by a reduction in dissolved oxygen and a slight disruption of nitrifying activity [33], evidenced by the reduction in NH_4_^+^, between the RFS outlet compared with its inlet and by the presence of some NO_2_^−^ (Table 3). The sump, being the point in the system with the lowest elevation, was naturally prone to collect solids being transported through the pipelines of the system by gravity. Elevated TOC concentration in the sump implied the presence of organic matter originating from dead plant material on the hydroponic table. The lowest TOC concentrations were found in the hydroponic table, indicating the consumption of C or loss of carbon dioxide to the environment.

Originating from the fish feces and uneaten feed, organic N is microbially mineralized to the N_inorg_ compounds [34, 35], which are of major importance for plant nutrition [36] but can also affect fish welfare [4]. In the aerobic part of the system, NO_3_^−^ represented the major part of N_inorg_, while in the anaerobic digester, NH_4_^+^ was the primary N compound (Table 3). Furthermore, the RFS showed higher values of NH_4_^+^ at its intake, but lower values at the outlet, suggesting both protein breakdown and some nitrification occurring in this part of the system.

Next to the chemical analysis of the different compartments of the system, we have chosen to use community profiling by T-RFLP [37] as a simple but reliable way to assess the bacterial and archaeal community. This method yields a first view on differences in the diversity of the two populations. The α-diversity indices indicated that the bacterial diversity was generally high, with slightly lower diversity in the anaerobic digester (Fig. 2) as sludge degradation is carried by specialized consortia of organisms [38]. The observed values for the Shannon index were lower (Fig. 2) as determined by amplicon sequencing before [21, 39], acknowledging that for T-RFLP, it is known that only the most abundant part of the community can be assessed [37, 40, 41]. However, the currently available studies that used amplicon sequencing only sampled a limited number of compartments [6, 21, 39], or even mainly focused on the biofilter only [42].

To our knowledge, archaeal communities have not been studied in detail in aquaponics systems [42], even though archaea can also be involved in N conversion processes [43, 44]. This study shows a considerable archaeal diversity in the biofilm samples from all compartments. However, a conclusion on the number of archaea in the different compartments nor of their identity cannot be derived from the community profiles.

The results of this study add up to the understanding of the nitrogen cycling functions of separate compartments of aquaponic systems. This can help designers, engineers and operators to optimize existing designs and configurations. By utilizing the nitrification capacity of plant rearing compartments, the size and cost of biofilters can be reduced. The operation of sludge reactors at different redox conditions is able to denitrify water and produce methane simultaneously, while fine tuning of methane production in anaerobic digesters allows controlling the abundance of methanogens. At the same time, system optimization would prevent undesired environmental conditions, which can lead to poor system performance.

## Conclusions

The findings of this project can confirm that there is a marked difference between aerobic and anaerobic biofilms in microbial community structure. The aerobic loop of the system, where most of the nitrification takes place, is characterized as being highly biodiverse [6, 21], while biofilms in the anaerobic loop, where nitrate, iron and sulfate reduction can take place, contain a more specialized and less diverse community. The connection point between the aerobic and the anaerobic loop of the system, the RFS, showed common conditions where both aerobic and anaerobic processes take place. In the future, a more detailed characterization of the microbial communities using next-generation sequencing and quantitative PCR would be required to determine which species and their corresponding genes are responsible for different processes in the aquaponic system.

## Methods

Three parallel running aquaponic systems, stocked with Nile tilapia (*Oreochromis niloticus*, with a stocking density of 10 kg m^− 3^) obtained from Til-Aqua International, the Netherlands, and various plants, were in constant operation since May 2017. Fish were healthy and fed ad libitum with a vegetarian feed, Tilapia Vegi, 3.0 mm (Hokovit, Hofmann Nutrition AG, Bützberg, Switzerland). Between May 2018 and November 2018, a mixture of 63 plants (basil - 41% (*Ocimum basilicum*), mint - 24% (*Mentha spicata*), melissa - 16% (*Melissa officinalis*), purslane - 5% (*Portulaca oleracea*), shiso - 4% (*Perilla frutescens*), asparagus pea - 3% (*Psophocarpus tetragonolobus*), oregano - 3% (*Origanum vulgare*), parsley - 2% (*Petroselinum crispum*), sorrel - 2% (*Rumex acetosa*) and salvia - 1% (*Salvia officinalis*)) were planted in all three systems. Beneficial organisms (*Encarsia formosa*, *Ichneumonidae* as Basil Protect, *Amblyseius swirskii*, *Amblyseius californicus* and *Chrysoperla carnea* obtained from Andermatt Biocontrol AG, Grossdietwil, Switzerland) were used for additional phyllosphere protection of the plants. The free software HydroBuddy [45] was used to calculate the weekly amount of Iron DTPA and Multi Micro Mix (Ökohum GmbH, Herrenhof, Switzerland), which were added directly to the hydroponic table of the system to provide the essential nutrients for the plants which could not be provided with the fish feed. Sampling took place in September 2018. During this time, two microbial and chemical samplings were performed. The system was operated for eight more months afterwards.

### System design

Each AP (Fig. 1), with a total volume of 4.3 m^3^, consisted of a fish tank, a solids removal unit (drum filter), solids thickening unit (RFS), a moving bed biofilter with biochips, a UV treatment zone, an oxygenation zone, a sump and 9 m^2^ hydroponic unit with a table raft system (Dryhydroponics BV, ‘s-Gravenhage, The Netherlands) floating on 25 mm of water. The system was complemented with an off-line anaerobic digester.

Temperature, dissolved oxygen, pH and electrical conductivity were continuously measured in the fish tank and logged with a LINN operating system (LINN Gerätebau GmbH, Lennestadt-Oedingen, Germany). System water temperature was maintained via a heat exchanger in the biofilter at 27 ± 2 °C and the oxygen level was kept at 100% saturation.

### Chemical and microbial analyses

Water samples were taken parallel to the microbial samples in nine locations throughout the system as indicated in Fig. 1. Water samples were analyzed for temperature, pH, electrical conductivity, oxygen saturation, redox potential, TOC, TN, NH_4_^+^, NO_2_^−^, NO_3_^−^, calcium, magnesium, sodium, potassium and sulfate (Additional file 1: Table S2). Biofilm samples were taken in triplicates throughout the system (Fig. 1). Surface biofilm samples were collected by scraping approximately 100 cm^2^ of biofilm from the surface using cotton swabs, while biofilm samples from the biofilter were obtained by collecting 20 biochips in a 50 mL Falcon tube (Additional file 1: Table S3). After sampling, the samples were immediately stored in a polystyrene box containing cooling elements until the end of the sampling and then stored at − 20 °C until further analysis.

### Microbial sample preparation and DNA extraction

Microbial biomass was obtained by adding ultrapure water to the biofilm samples, vortexing the tubes for 1 min, followed by 5 min in an ultrasonic bath at room temperature (Sonorex, Bandelin, Berlin, Germany). The tubes were then vortexed for an additional 2 min, followed by 10 min in the ultrasonic bath. Subsequently, biochips or cotton swabs were removed using a pincer. Microbial biomass was collected as a pellet after centrifugation (5000 rpm, 10 min). The pellets were used for further DNA extractions. All samples were extracted with the DNeasy PowerSoil Kit (Qiagen, Venlo, The Netherlands) according to the manufacturer’s instructions. After the extraction, samples were stored at − 20 °C until further analysis.

### Microbial sample analyses

The partial 16S rRNA gene was amplified from DNA extractions by PCR using fluorescently labeled primers for bacteria and archaea (Additional file 1: Table S4). The DNA Polymerase KAPA2G Robust HotStart ReadyMix (Sigma-Aldrich, Missouri, United States) was used within a suitable master-mix according to manufacturer’s instructions. PCR amplifications were carried out on a T100 Thermocycler (Bio-Rad Laboratories, Inc., Hercules, California, United States). Products of the PCR were end-treated for the correction of the overhanging ends effect [37] and were cleaned with a Millipore MultiScreen PCR_μ96_ filter plate (Merck KGaA, Darmstadt, Germany). Finally, the products were resuspended in 25 μL ddH_2_O. Purified PCR amplicons were digested by using the restriction enzyme *Alu*I according to the manufacturer’s instructions. Each 1 μL of digestion product was mixed with 18.65 μL Hi-Di formamide and 0.35 μL GeneScan LIZ 600 Size Standard (Thermofisher Scientific™, Massachusetts, United States), denatured and analyzed using ABI 3500 capillary sequencer (Thermofisher Scientific™).

### Data analyses

Profiles obtained with T-RFLP were analyzed using the GeneMapper® Software 5 (Applied Biosystems, Thermofisher Scientific, Massachusetts, United States). Restriction fragments between 40 and 700 base pairs were included in the analysis and exported as raw data. Further data processing was carried out using the software T-REX [46] and PAST 3.24 [47]. All statistical analyses and graphics were carried out with R statistical software version 3.5.2 [48] and packages “agricolae” [49], “devtools” [50], “dplyr” [51], “ggbiplot” [52], “ggplot2” [52], “ggpubr” [53], “moments” [54], “scales” [55] and “vegan” [56]. To test for differences, Kruskal-Wallis rank-sum test based on Fisher’s LSD test with a significance level of α = 5%. Principal component analysis was used to test the main abiotic factors influencing the variance between the compartments of the system. The analysis of the microbial communities was done separately for bacteria and archaea using T-RF peak area. To characterize microbial diversity, Shannon and Simpson diversity indices were calculated [57, 58]. Non-metric multidimensional scaling was used to analyze shifts in the community between the compartments. Additionally, generalized additive models were fitted onto bacterial and archaeal NMDS to assess the potential influence of abiotic parameters on the community structure.

## Supplementary Information

**Additional file 1. **The file contains all supplementary tables and figures listed below. **Table S1.** Shannon and Simpson diversity indices on different sampling times. **Table S2.** Chemical sampling, measured parameters, sample preparation and further analysis. **Table S3.** Microbial biofilm sampling and used material at the sampling and further sample preparation. **Table S4.** Primers used for the microbial analysis. **Figure S1.** Non-metric multidimensional scaling plot of bacterial and archaeal communities with 95% confidence eclipses in different compartments of the aquaponic system. **Figure S2.** Principal component analysis (PCA) with 95% confidence eclipses of measured environmental parameters. **Figure S3.** Non-metric multidimensional scaling plot of bacterial community with generalized additive models (gray lines) of fitted environmental variables. **Figure S4.** Non-metric multidimensional scaling plot of archaeal community with generalized additive models (gray lines) of fitted environmental variables. **Figure S5.** Redundancy analysis (RDA) of the bacterial (A) and archaeal (B) community in different compartments of the aquaponic system.

## Data Availability

The datasets used and/or analyzed during the current study are available from the corresponding author on reasonable request.

## Abbreviations

C: Carbon
N: Nitrogen
NH_4_^+^: Ammonium
NMDS: Non-metric multidimensional scaling
NO_2_^−^: Nitrite
NO_3_^−^: Nitrate
N_org_: Organic nitrogen
OTU: Operational taxonomic units
RFS: Radial flow settler
TN: Total nitrogen
TOC: Total organic carbon
T-RFLP: Terminal restriction fragment length polymorphism
