# Supplementary material for: Environmental parameters and microbial community profiles as indication towards microbial activities and diversity in aquaponic system compartments
Source: BMC Microbiol. 2021 Jan 6;21:12. doi: 10.1186/s12866-020-02075-0 (PMC7789318; doi:10.1186/s12866-020-02075-0)
Supplement: Supplementary file 1 — Additional file 1. The file contains all supplementary tables and figures listed below. Table S1. Shannon and Simpson diversity indices on different sampling times. Table S2. Chemical sampling, measured parameters, sample preparation and further analysis. Table S3. Microbial biofilm sampling and used material at the sampling and further sample preparation. Table S4. Primers used for the microbial analysis. Figure S1. Non-metric multidimensional scaling plot of bacterial and archaeal communities with 95% confidence eclipses in different compartments of the aquaponic system. Figure S2. Principal component analysis (PCA) with 95% confidence eclipses of measured environmental parameters. Figure S3. Non-metric multidimensional scaling plot of bacterial community with generalized additive models (gray lines) of fitted environmental variables. Figure S4. Non-metric multidimensional scaling plot of archaeal community with generalized additive models (gray lines) of fitted environmental variables. Figure S5. Redundancy analysis (RDA) of the bacterial (A) and archaeal (B) community in different compartments of the aquaponic system. [file 12866_2020_2075_MOESM1_ESM.pdf]

SUPPORTING MATERIAL

Environmental parameters and microbial community profiles as indication towards microbial activities and diversity in aquaponic system compartments

Zala Schmautz [1,2], Carlos A. Espinal [3], Andrea M. Bohny [2], Fabio Rezzonico [4], Ranka Junge [1], Emmanuel Frossard [2], Theo H. M. Smits [4,*]

[1] Ecological Engineering Centre, Institute of Natural Resource Sciences, Zurich University of Applied Sciences, Wädenswil, Switzerland.

[2] Group of Plant Nutrition, Institute of Agricultural Sciences, ETH Zurich, Lindau, Switzerland.

[3] Landing Aquaculture, Boxtel, The Netherlands

[4] Environmental Genomics and Systems Biology Research Group, Institute of Natural Resource Sciences, Zurich University of Applied Sciences, Wädenswil, Switzerland.

[*] Correspondence: theo.smits@zhaw.ch

Content

Table S1. Shannon and Simpson diversity indices on different sampling times.

Table S2. Chemical sampling, measured parameters, sample preparation and further analysis.

Table S3. Microbial biofilm sampling and used material at the sampling and further sample preparation.

Table S4. Primers used for the microbial analysis.

Figure S1. Non-metric multidimensional scaling plot of bacterial and archaeal communities with 95% confidence eclipses in different compartments of the aquaponic system.

Figure S2. Principal component analysis (PCA) with 95% confidence eclipses of measured environmental parameters.

Figure S3. Non-metric multidimensional scaling plot of bacterial community with generalized additive models (gray lines) of fitted environmental variables.

Figure S4. Non-metric multidimensional scaling plot of archaeal community with generalized additive models (gray lines) of fitted environmental variables.

Figure S5. Redundancy analysis (RDA) of the bacterial (A) and archaeal (B) community in different compartments of the aquaponic system

Supplementary tables

Table S1. Shannon and Simpson diversity indices on different sampling times presented as mean ± SEM, where letters present significant differences based on Kruskal-Wallis rank sum test followed by Fisher's LSD test (α = 5%) between different compartments of the aquaponic system (fish tank, biofilter, sump, hydroponic table, RFS inflow, RFS outflow and anaerobic digester). Numbers in the brackets present number of the samples.

		Week	Fish tank	Biofilter	Sump	HP table	RFS inflow	RFS outflow	Digester
Shannon diversity	Bacteria	37	3.85 ± 0.04 a (18)	3.80 ± 0.05 ab (8)	3.81 ± 0.09 a (9)	3.85 ± 0.06 a (9)	3.75 ± 0.04 ab (8)	3.65 ± 0.10 ab (9)	3.45 ± 0.10 b (9)
		39	3.95 ± 0.04 c (18)	4.10 ± 0.06 ab (8)	4.05 ± 0.04 bc (9)	4.23 ± 0.04 a (9)	3.97 ± 0.04 c (9)	3.96 ± 0.05 c (9)	3.56 ± 0.08 d (9)
		37 and 39	3.90 ± 0.03 ab (36)	3.95 ± 0.05 ab (18)	3.93 ± 0.05 ab (18)	4.04 ± 0.06 a (18)	3.87 ± 0.04 b (17)	3.80 ± 0.07 b (18)	3.51 ± 0.06 c (18)
	Archaea	37	1.73 ± 0.20 a (7)	1.82 ± 0.25 a (3)	1.25 ± 0.09 a (5)	0.87 ± 0.18 a (2)	1.56 ± 0.29 a (4)	1.83 ± 0.42 a (3)	2.03 ± 0.07 a (4)
		39	2.65 ± 0.08 a (18)	2.32 ± 0.19 a (6)	2.72 ± 0.09 a (9)	2.77 ± 0.10 a (8)	2.71 ± 0.10 a (9)	2.76 ± 0.1 0 a (9)	1.98 ± 0.26 a (2)
		37 and 39	2.39 ± 0.11 a (25)	2.16 ± 0.17 a (9)	2.19 ± 0.21 a (14)	2.39 ± 0.27 a (10)	2.35 ± 0.18 a (13)	2.53 ± 0.17 a (12)	2.01 ± 0.08 a (6)
Simpson diversity	Bacteria	37	0.04 ± 0.00 b (18)	0.04 ± 0.00 ab (8)	0.04 ± 0.00 b (9)	0.04 ± 0.00 b (9)	0.04 ± 0.00 b (8)	0.05 ± 0.00 ab (9)	0.06 ± 0.01 a (9)
		39	0.04 ± 0.00 b (18)	0.03 ± 0.00 bcd (8)	0.03 ± 0.00 cd (9)	0.03 ± 0.00 d (9)	0.04 ± 0.00 bc (9)	0.04 ± 0.00 bcd (9)	0.06 ± 0.00 a (9)
		37 and 39	0.04 ± 0.00 b (36)	0.04 ± 0.00 de (16)	0.04 ± 0.00 cd (18)	0.03 ± 0.00 e (18)	0.04 ± 0.00 bc (17)	0.04 ± 0.00 b (18)	0.06 ± 0.00 a (18)
	Archaea	37	0.22 ± 0.05 a (7)	0.24 ± 0.02 a (3)	0.31 ± 0.03 a (5)	0.43 ± 0.07 a (2)	0.25 ± 0.05 a (4)	0.22 ± 0.08 a (3)	0.16 ± 0.01 a (4)
		39	0.10 ± 0.01 ab (18)	0.17 ± 0.03 a (6)	0.10 ± 0.01 ab (9)	0.09 ± 0.01 ab (8)	0.10 ± 0.02 b (9)	0.09 ± 0.01 b (9)	0.22 ± 0.04 a (2)
		37 and 39	0.14 ± 0.02 a (25)	0.19 ± 0.02 a (9)	0.18 ± 0.03 a (14)	0.16 ± 0.05 a (10)	0.15 ± 0.03 a (13)	0.12 ± 0.03 a (12)	0.18 ± 0.02 a (6)

Table S2. Chemical sampling, measured parameters, sample preparation and further analysis.

Parameters	Where?	Sample preparation	Lab equipment	Company
**pH** [-], **T** [°C]	Directly on the sampling spot	/	Probe PHC10103 & HQ40d portable multimeter	Hach Lange, Loveland, Colorado, United States
**Redox potential** [mV]	On spot	/	Probe MTC10101 & HQ40d portable multimeter	Hach Lange, Loveland, Colorado, United States
**El. conductivity** [µS cm^{-1}]	On spot	/	Probe CDC40103 & HQ40d portable multimeter	Hach Lange, Loveland, Colorado, United States
**Oxygen saturation** [%]	On spot	/	Probe LDO10101 & HQ40d portable multimeter	Hach Lange, Loveland, Colorado, United States
**NH_4^+-N** [mg L^{-1}]	Stored in 50 mL falcon tube, laboratory	Filtered, 0.45 µm	Cuvette test LCK304 & DR 3800™ Benchtop Spectrophotometer	Hach Lange, Loveland, Colorado, United States
**NO_2^--N** [mg L^{-1}]	Stored in 50 mL falcon tube, laboratory	Filtered, 0.45 µm	Cuvette test LCK341 & DR 3800™ Benchtop Spectrophotometer	Hach Lange, Loveland, Colorado, United States
**NO_3^--N** [mg L^{-1}]	Stored in 50 mL falcon tube, laboratory	Filtered, 0.45 µm	Cuvette test LCK339 & DR 3800™ Benchtop Spectrophotometer	Hach Lange, Loveland, Colorado, United States
**Ca^{2+}, Mg^{2+}, K^+** [mg L^{-1}]	Stored in 15 mL falcon tube, laboratory	Filtered, 0.22 µm, 1 µL 2M HNO_3 per 1 mL sample	Ion chromatograph, 930, Compact IC flex	Methrom Schweiz AG, Zofingen, Switzerland
**Cl^-, SO_4^{2-}** [mg L^{-1}]	Stored in 15 mL falcon tube, laboratory	Filtered, 0.22 µm	Ion chromatograph, 930, Compact IC flex	Methrom Schweiz AG, Zofingen, Switzerland
**Total nitrogen, total organic carbon** [mg L^{-1}]	Stored in 50 mL falcon tube, laboratory	High solid content samples were homogenized (Tissue-Tearor, Biospec, Bartlesville, USA).	TOC and TN-Analyzer	TOC-L and TNM-L, Shimadzu, Kyoto, Japan)

Table S3. Microbial biofilm sampling and used material at the sampling and further sample preparation.

Microbial biofilm sample	Material used at the sampling	Material used for further sample preparation
Fish tank wall, sump	Cotton swab, stencil (100 cm^2), 2 mL tube	1.5 mL of ultrapure water
Biochips	20 biochips, 50 mL Falcon tube	40 mL of ultrapure water
Hydroponic table, radial flow settler – inflow and outflow, anaerobic digester	Cotton swab (approx. 100 cm^2), 2 mL tube	1.5 mL of ultrapure water

Table S4. Primers used for the microbial analysis (Microsynth AG, Balgach, Switzerland).

Organism	Primer	Annealing temperature	Amplicon length	Fluorophore	Source
Bacteria	8F_Red: 5'-AGA GTT TGA TCC TGG CTC AG-3'	59°C	416 - 560	AT565	[1]
	534R_Green: 5'-ATT ACC GCG GCT GCT GGC-3'			AT532	[2]
Archaea	ARCH109f_Green: 5'-ACK GCT CAG TAA CAC GT-3'	55°C	832 - 880	AT532	[3]
	ARCH958r_Blue: 5'-YCC GGC GTT GAM TCC AAT T-3'			FAM	[4]

Sources:

1. Lane DJ. 16S/23S rRNA sequencing. In: Nucleic Acid Techniques in Bacterial Systematics, ed. E. Stackebrandt and M. Goodfellow. New York: John Wiley & Sons.; 1991. p. 115–75.
2. Muyzer G, Waal EC de, Uitterlinden AG. Profiling of complex microbial populations by denaturing gradient gel electrophoresis analysis of polymerase chain reaction-amplified genes coding for 16S rRNA. Appl Environ Microbiol. 1993;59:695–700.
3. Großkopf R, Janssen PH, Liesack W. Diversity and structure of the methanogenic community in anoxic rice paddy soil microcosms as examined by cultivation and direct 16S rRNA gene sequence retrieval. Appl Environ Microbiol. 1998;64:960–9.
4. Luna GM, Stumm K, Pusceddu A, Danovaro R. Archaeal diversity in deep-sea sediments estimated by means of different terminal-restriction fragment length polymorphisms (T-RFLP) protocols. Curr Microbiol. 2009;59:356–61.

Supplementary figures

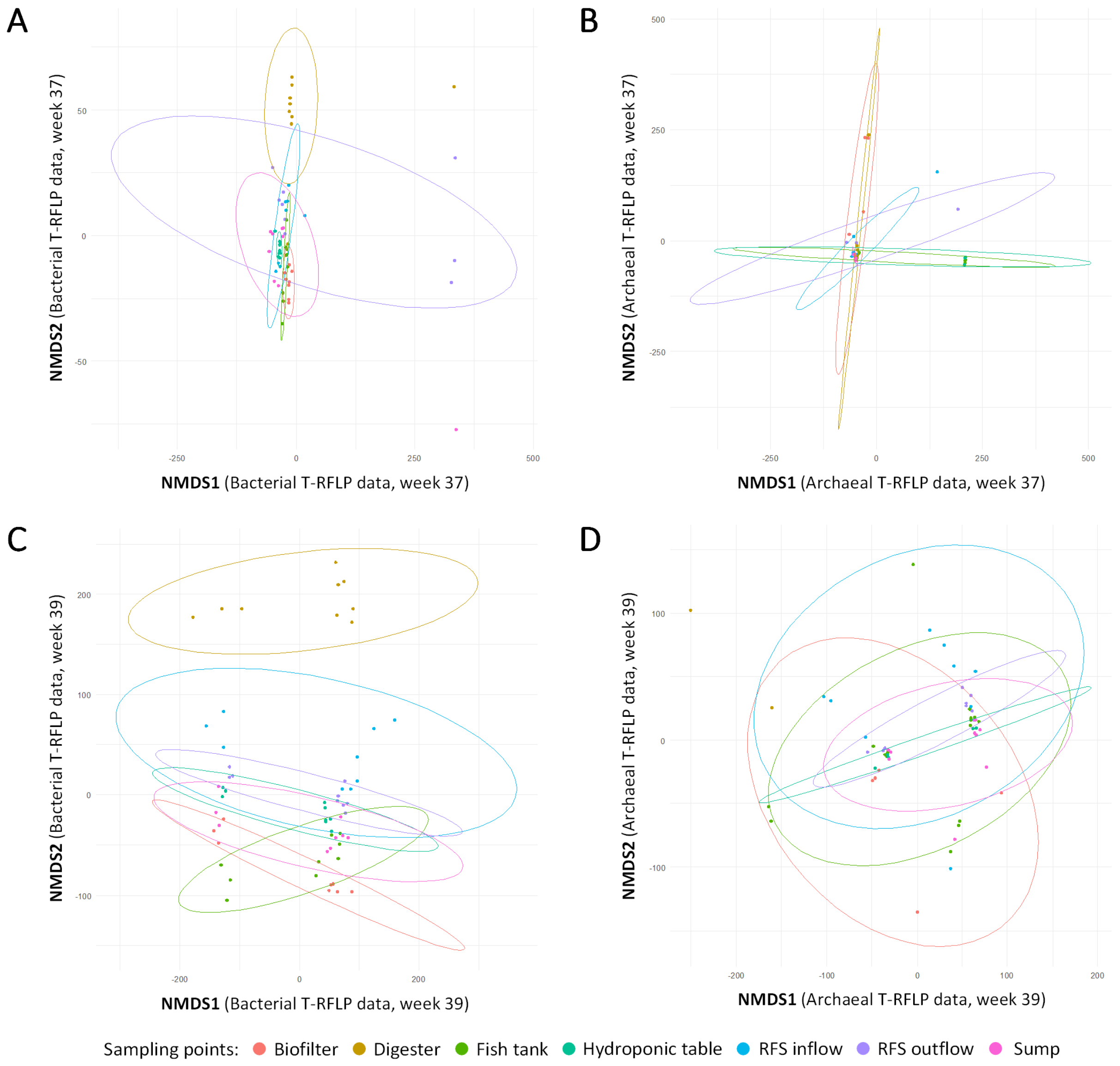

Figure S1. Non-metric multidimensional scaling plot of bacterial (A – week 37, ADONIS $R^2 = 0.403$, dimensions = 2, stress = 0.081 and C – week 39, ADONIS $R^2 = 0.413$, dimensions = 2, stress = 0.150) **and archaeal** (B – week 37, ADONIS $R^2 = 0.225$, dimensions = 2, stress = 0.066 and D – week 39, ADONIS $R^2 = 0.298$, dimensions = 2, stress = 0.124) **communities with 95% confidence eclipses in different compartments of the aquaponic system** (fish tank, biofilter, sump, hydroponic table, RFS inflow, RFS outflow and anaerobic digester).

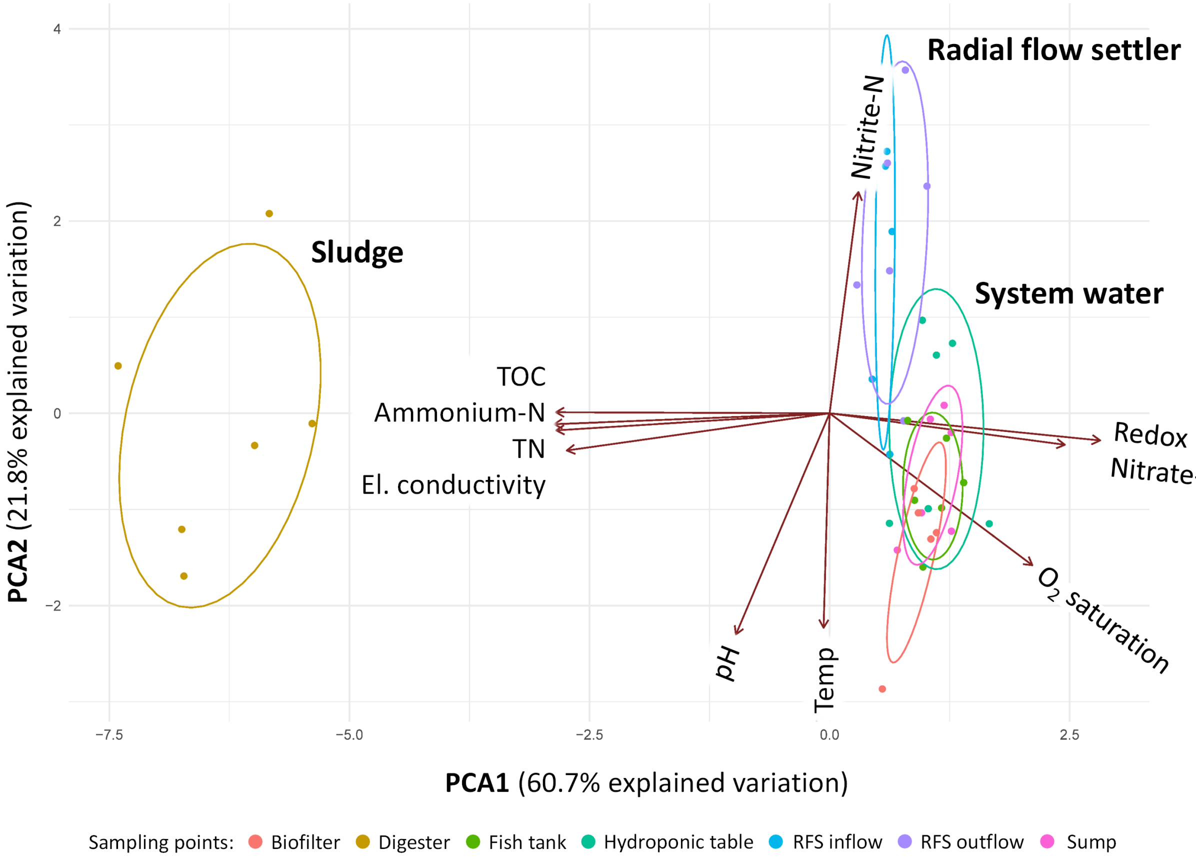

Figure S2. Principal component analysis (PCA) with 95% confidence eclipses of measured environmental parameters (temperature, pH, electrical conductivity, dissolved oxygen, redox potential, total nitrogen and total organic carbon, ammonium-nitrogen, nitrite-nitrogen, nitrate-nitrogen) explaining 82.5% of data variance in different compartments of the aquaponic system (fish tank, biofilter, sump, hydroponic table, RFS inflow, RFS outflow and anaerobic digester).

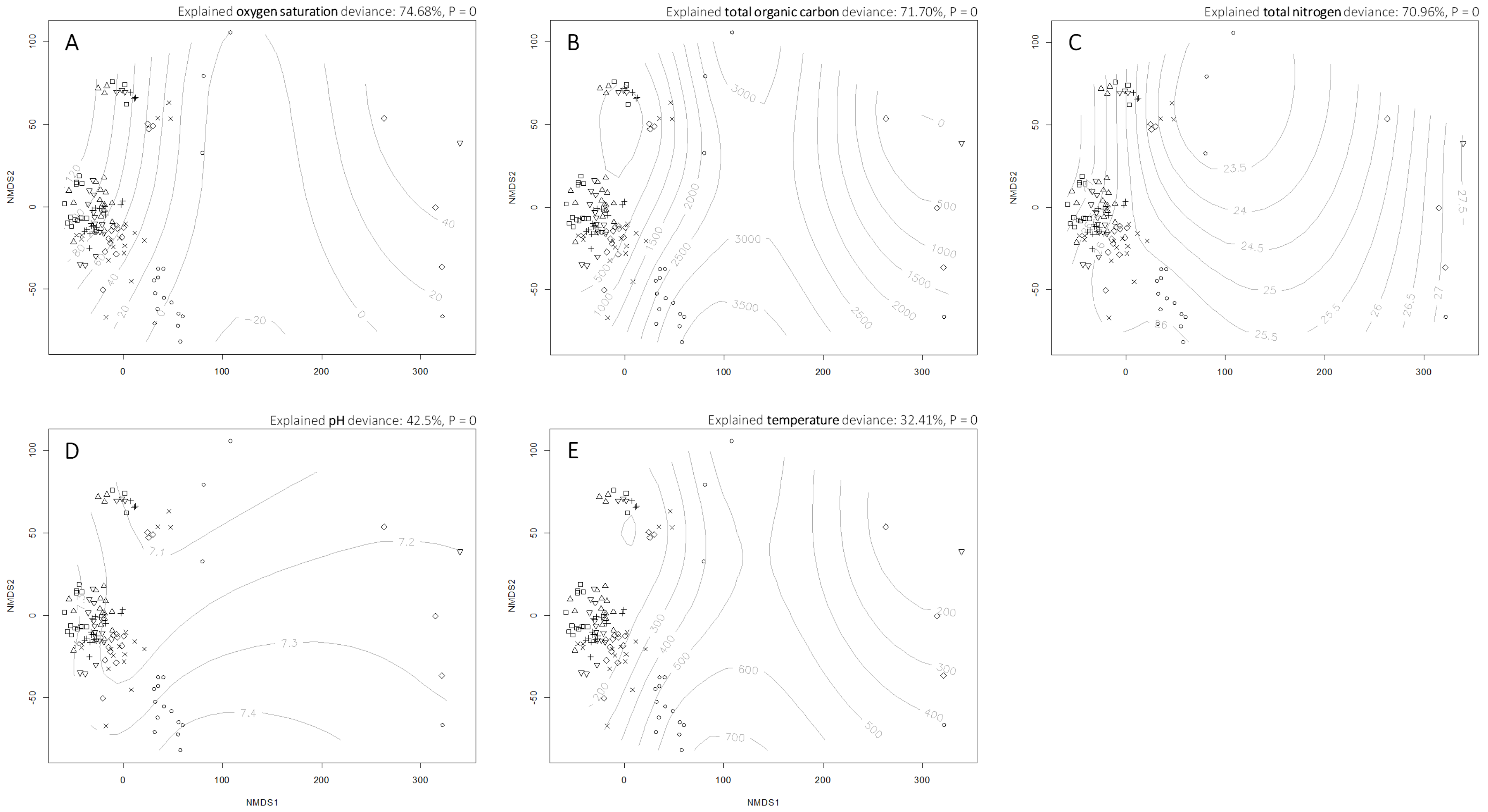

Sampling points: □ Biofilter ○ Digester △ Fish tank + Hydroponic table × RFS inflow ◇ RFS outflow ▽ Sump

Figure S3. Non-metric multidimensional scaling plot of bacterial community with generalized additive models (gray lines) of fitted environmental variables (A, oxygen saturation; B, total organic carbon; C, total nitrogen; D, pH; E, temperature) in different compartments of the aquaponic system (fish tank, biofilter, sump, hydroponic table, RFS inflow, RFS outflow and anaerobic digester).

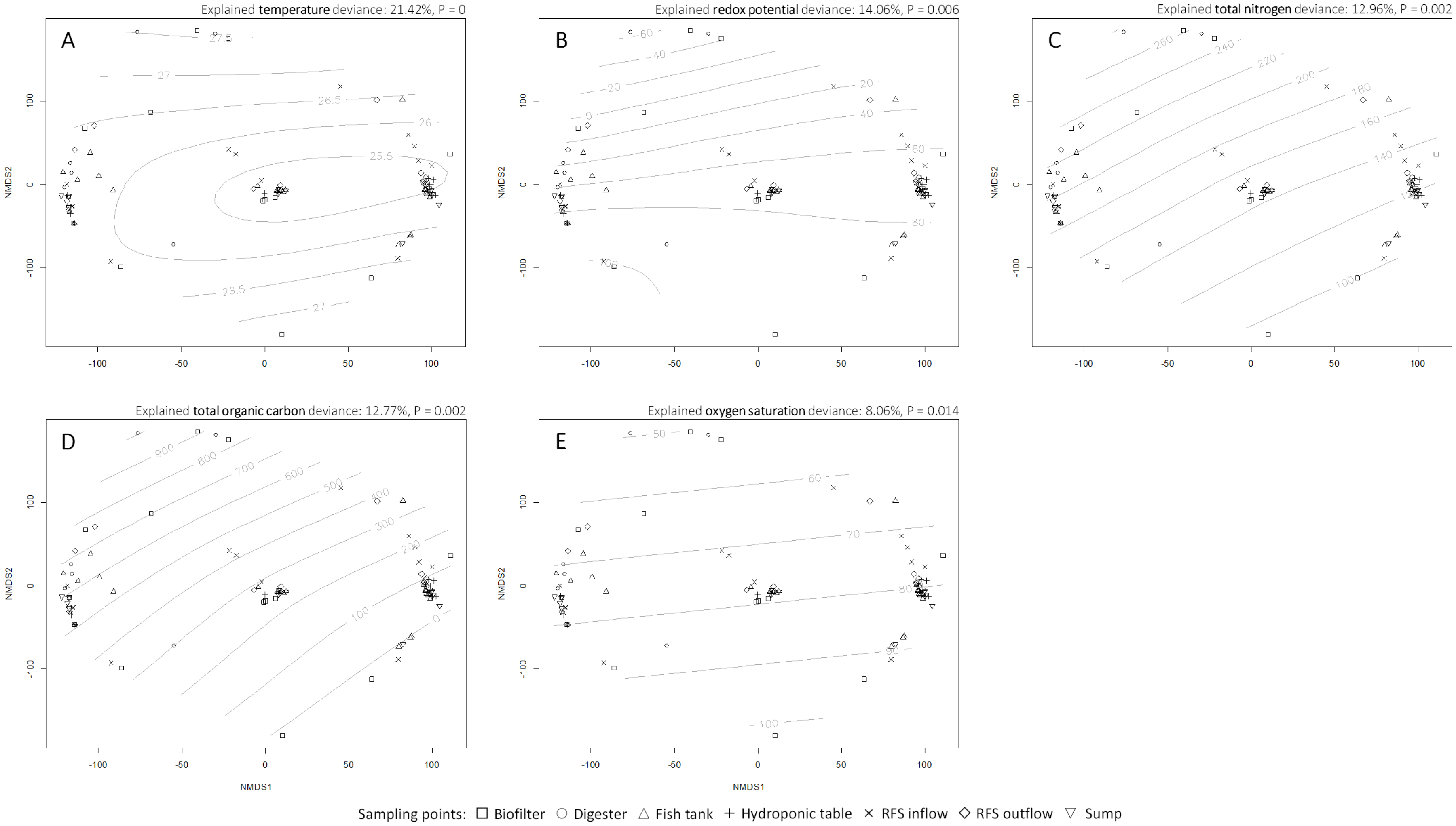

Figure S4. Non-metric multidimensional scaling plot of archaeal community with generalized additive models (gray lines) of fitted environmental variables (A, temperature; B, redox potential; C, total nitrogen; D, total organic carbon; E, oxygen saturation) in different compartments of the aquaponic system (fish tank, biofilter, sump, hydroponic table, RFS inflow, RFS outflow and anaerobic digester).

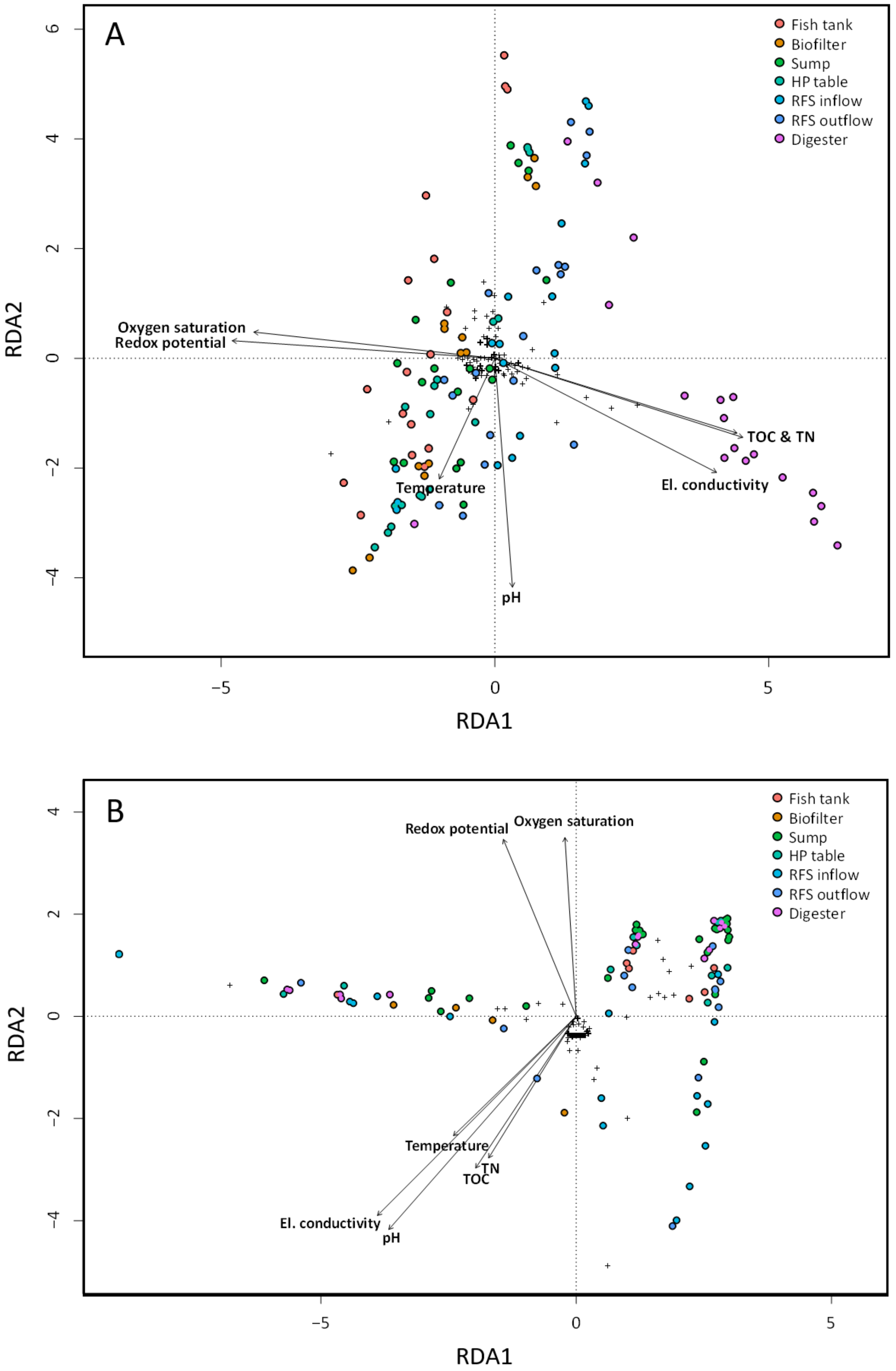

Figure S5. Redundancy analysis (RDA) of the bacterial (A) and archaeal (B) community in different compartments of the aquaponic system (fish tank, biofilter, sump, hydroponic table, RFS inflow, RFS outflow and anaerobic digester) with environmental parameters (redox potential, oxygen saturation, electrical conductivity, pH, temperature, total nitrogen and total organic carbon).
